# Antipsychotic Prescribing Patterns in Patients with Dementia Attending a Psychogeriatric Clinic in Sri Lanka

**DOI:** 10.1192/bjo.2026.11096

**Published:** 2026-06-30

**Authors:** Amodha Medagedara, LD Manchanayake, MGN Priyangani, DVM Dias, Kapila Ranasinghe

**Affiliations:** 1 ELFT, Luton, United Kingdom; 2 Faculty of Medicine, University of Kelaniya, Ragama, Sri Lanka; 3 NIMH, Angoda, Sri Lanka

## Abstract

**Aims:**

Usage of antipsychotic medications in patients with dementia is associated with heightened incidence of adverse effects and mortality. Yet their prescription is on the rise to contain behavioural and psychological symptoms of dementia (BPSD). Thus the aim of thisstudy is to as certain patterns in antipsychotic prescribing over the years from 2019 to January 2025 among patients with dementia attending Deeghayu psychogeriatric clinic, at the National Institute of Mental Health, Angoda, the largest psychogeriatric clinic facility in Sri Lanka.

**Methods:**

A descriptive cross-sectional analysis of the data extracted from the patient records was done.

**Results:**

Total of 493 patients had been viewed including 306 (62%) with dementia. Alzheimer’s, vascular and mixed aetiologies had been the commonest with a prevalence of 124 (40.5%), 69 (22.5%) and 92 (30.6%) respectively. Out of them 228 (74.5%) had BPSD. Among them 172 (75.4%) had been initiated on antipsychotics. Quetiapine, risperidone, olanzapine and aripiprazole had been prescribed for 84 (48.8%), 16, 8 and 1 patients respectively and 63(36.6%) were on a combination of 2 antipsychotics out of which majority 45 (71.4%) were on the combination of risperidone and quetiapine. Among those who were not on antipsychotics for BPSD, 49 (21.5%) were on antidepressants for depression and 7 (3.1%) were solely on behavioural management. Poor sleep, wandering, aggression/agitation, hallucinations, disinhibition, delusions and mania had been the indication for prescription of antipsychotics in 107 (46.3%), 71 (30.7%), 80 (32.6%), 51(22%), 31(13.4%), 26 (11.6%) and 19 (8.2%) patients respectively. Percentage with two types of BPSD were 37.2% while 20.2% had more than two types of BPSD. Out of those with depression 34 (69.4%) had been prescribed antipsychotics out of which 27(79.4%) were on quetiapine.

**Conclusion:**

Antipsychotic prescription is of high prevalence. Identifying and addressing the underlying cause leading to BPSD and usage of non-pharmacological strategies as the firstline of management can minimize the usage of antipsychotics. Further research is warranted to evaluate tolerance and effectiveness of the prescribed antipsychotics.

